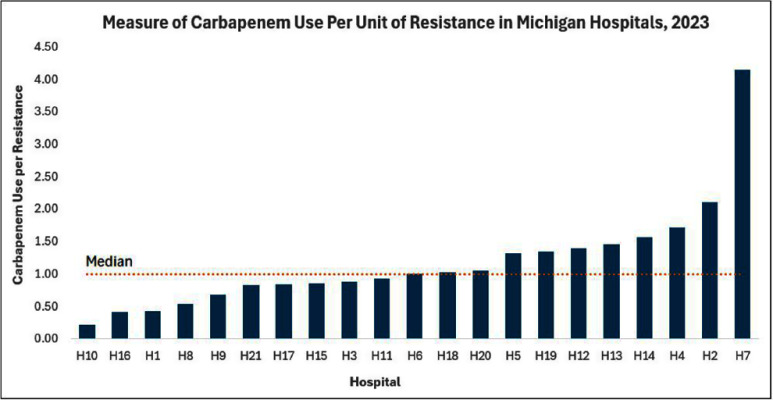# Linking Antimicrobial Use and Resistance Data to Obtain a Surrogate for Appropriateness: An Approach for Assessing Carbapenem Use

**DOI:** 10.1017/ash.2025.270

**Published:** 2025-09-24

**Authors:** Susan Catlin, Anne Haddad, Elisia Stier, Brenda Brennan, Anurag Malani, Jason Pogue

**Affiliations:** 1 Michigan Department of Health and Human Services; 2 Michigan Department of Health and Human Services; 3 Michigan Department of Health and Human Services; 4 Michigan Department of Health and Human Services; 5 Trinity Health Michigan; 6 University of Michigan College of Pharmacy

## Abstract

**Background:** Carbapenem use is a primary target of antimicrobial stewardship programs with attempts made to limit usage to patients with suspected or documented multidrug resistant Gram-negative infections. Antimicrobial use (AU) data and the standardized antimicrobial administration ratio (SAAR) are metrics for comparing observed to predicted days of antimicrobial therapy and are increasingly used by stewardship programs to assess use within their institutions. However, the SAAR does not account for individual drug class or resistance rates within an institution, limiting the ability to assess appropriateness and whether a high or low SAAR requires action. To try to assess carbapenem use as a function of resistance within hospitals in Michigan, we developed a novel measure utilizing National Healthcare Safety Network (NHSN) AU and Antimicrobial Resistance (AR) data. **Methods:** Included hospitals had reported both AU and AR data for the calendar year of 2023. To assess the “resistance burden” requiring carbapenem use at an institution, three antimicrobial resistance phenotypes from the NHSN AR data were chosen as a surrogate; extended-spectrum cephalosporin resistant (ESC) E.coli, ESC Klebsiella spp., and resistant Pseudomonas aeruginosa, which was defined as any isolate intermediate or resistant to cefepime, ceftazidime, or piperacillin/tazobactam. The resistance rates for these three phenotypes were combined to create a weighted antibiogram for total resistance burden requiring carbapenem usage at an institution. AU data for carbapenem use per 1,000 days present for each institution was then employed to normalize carbapenem use (numerator) to resistance burden (denominator at each institution), measured as carbapenem use per unit of resistance. The median value for included hospitals was then calculated, and finally, institutional use relative to this median was reported. **Results:** Twenty-one hospitals, ranging from 6 to 1,011 beds, met inclusion criteria. There were 18 acute care and 3 critical access hospitals; 19 (90.4%) were part of a health system. When normalized to the median value, adjusted carbapenem use per unit of resistance within hospitals ranged from 0.214 to 4.155 (Figure 1). The highest value of adjusted carbapenem use per resistance was 19.4 times that of the lowest value. **Conclusion:** This novel measure of antimicrobial use attempts to correct for the burden of resistance in individual facilities. As such, when applied to hospital populations, this represents a step forward in assessing antimicrobial use appropriateness and would have public health impact related to antimicrobial stewardship efforts. Future objectives include application to additional hospitals, years of data, antimicrobial resistance phenotypes, and agents.

**Figure:**